# Supplementary material for: The effect of helix-inducing constraints and downsizing upon a transcription block survival-derived functional cJun antagonist
Source: Cell Rep Phys Sci. 2022 Oct 19;3(10):101077. doi: 10.1016/j.xcrp.2022.101077 (PMC9582194; doi:10.1016/j.xcrp.2022.101077)
Supplement: Document S1. Figures S1–S6 [file mmc1.pdf]

Cell Reports Physical Science, Volume *3*

Supplemental information

The effect of helix-inducing constraints and downsizing upon a transcription block survival-derived functional cJun antagonist

Andrew Brennan, James T. Leech, Neil M. Kad, and Jody M. Mason

Supporting Information

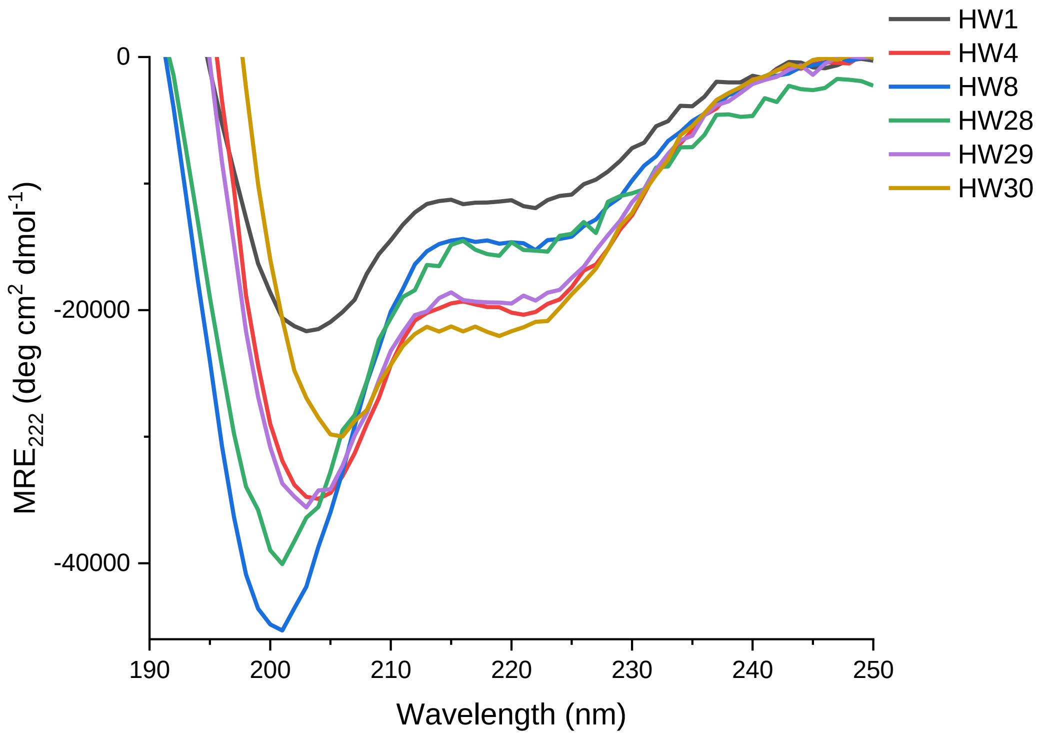

Figure S1 – Circular dichroism spectra showing the changes in peptide structure during the optimisation process. In general, this shows that N-terminal truncation increased helicity, C-terminal truncation decreased helicity and that lactamisation restored lost helicity.

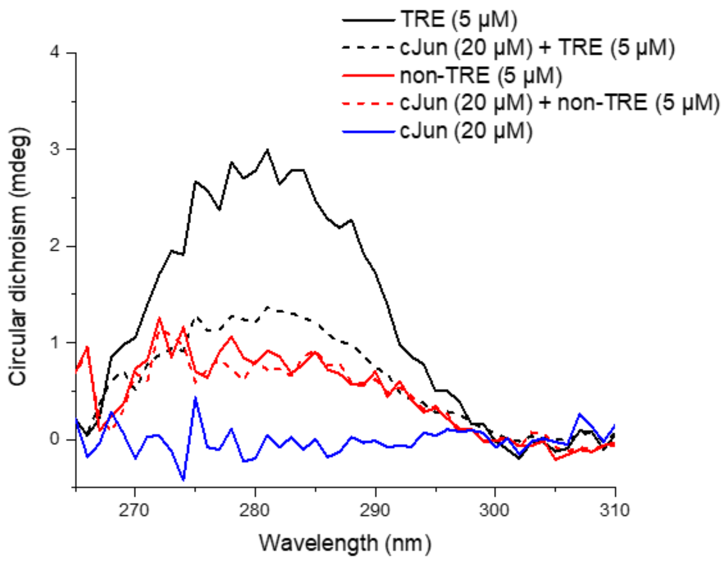

Figure S2 – Circular dichroism spectra showing the TRE site-specific shift in DNA structure caused by cJun binding.

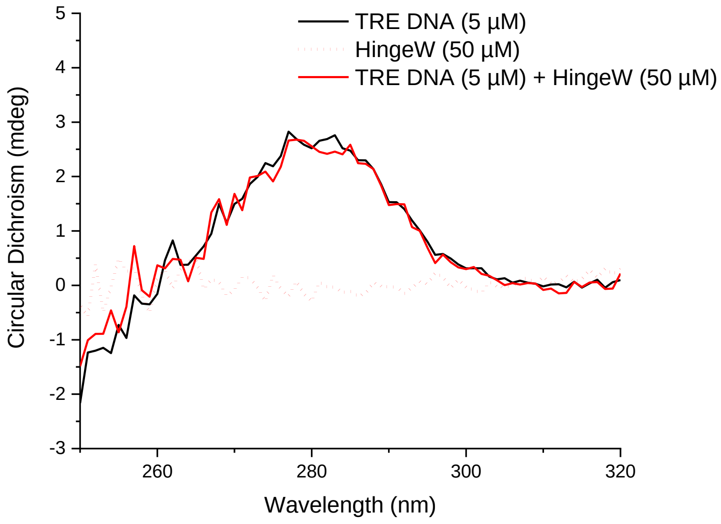

Figure S3 – HingeW does not interact with TRE DNA. Proteins do not absorb in this wavelength range, so the CD signal observed occurs due to the DNA structure, which is not perturbed upon addition of HingeW.

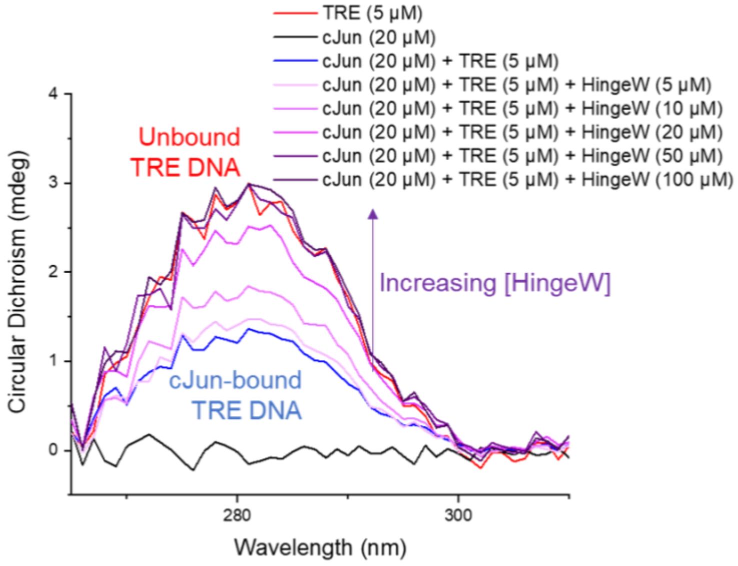

Figure S4 – CD spectra showing the TRE DNA peak shift at 281 nm induced by cJun binding, and its subsequent reversal by HingeW indicative of effective antagonism.

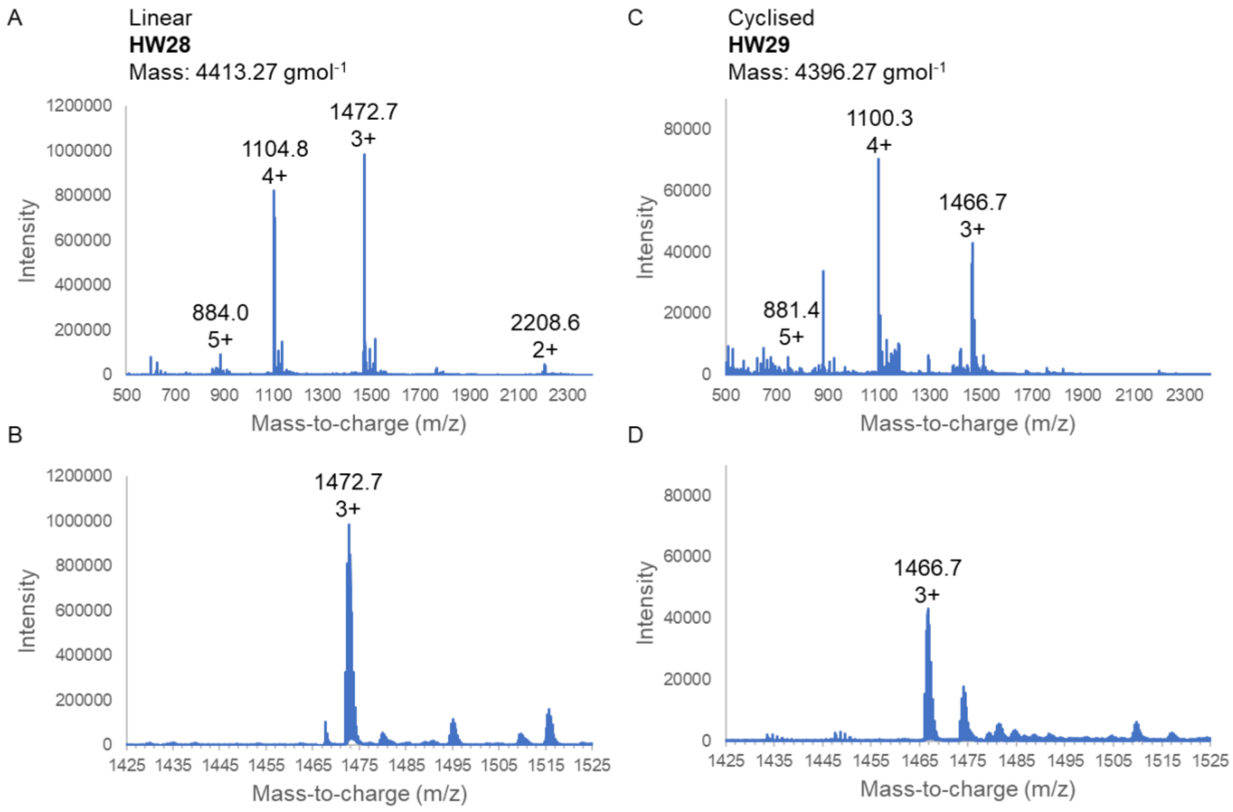

Figure S5 – Mass spectrometry was utilised to confirm peptide identity, to illustrate the shift in mass which occurs due to the loss of water during the lactamisation reaction. (A) MS spectrum of linear peptide HW28 and (B) an expanded region of the same spectrum to highlight the 3+ charge state. (C) MS spectrum of cyclised peptide HW29 and (D) an expanded region of the same spectrum to highlight the 3+ charge state. For this charge state a shift of 6 m/z occurs as predicted, as 6 Da x 3=18 Da (mass of water).

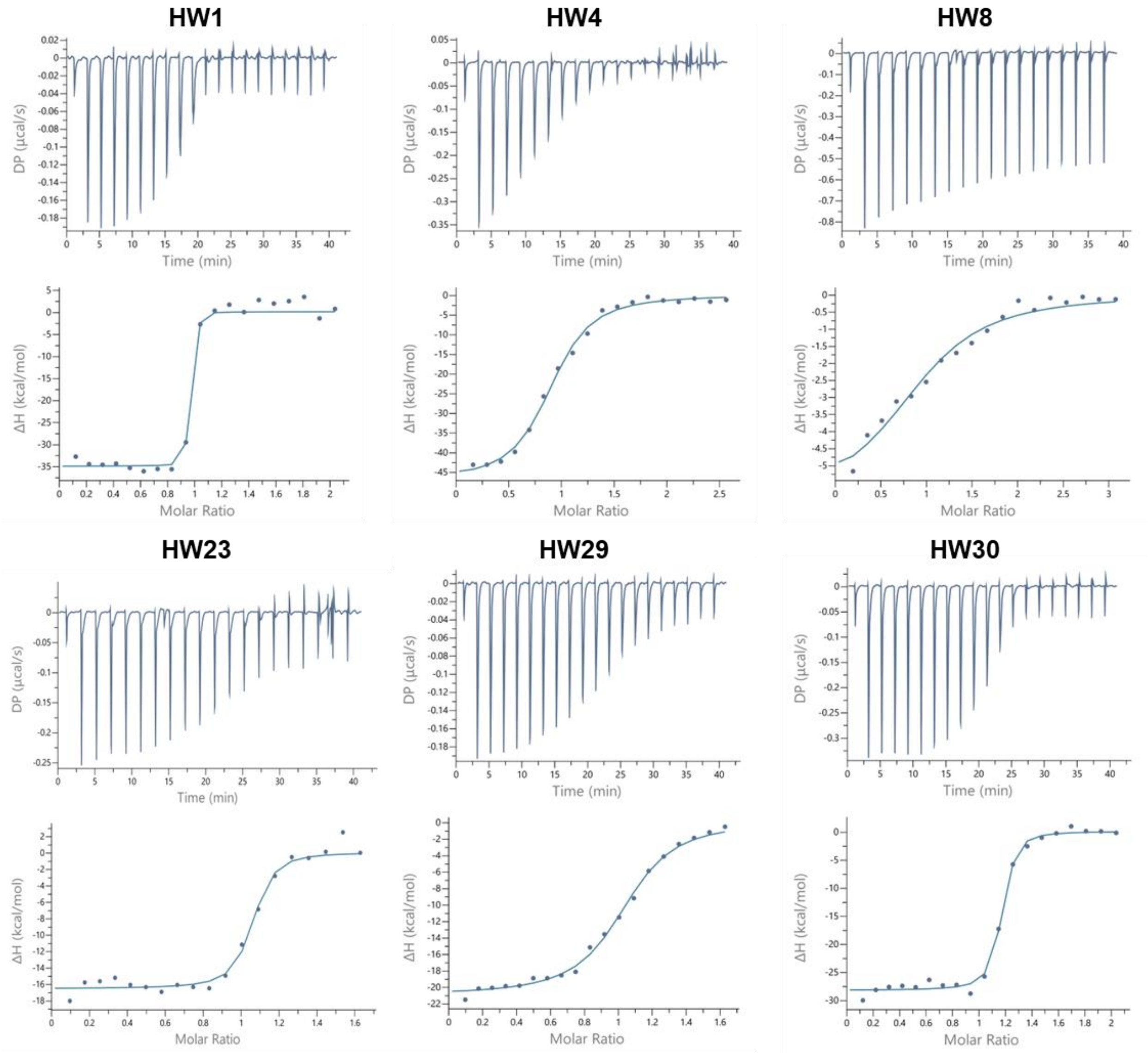

Figure S6 – Isothermal titration calorimetry data for the antagonist peptides binding to cJun. The raw power compensation plot is shown in the upper graph and the integrated data points and single site model fitted line are shown in the lower graph.
