# Supplementary material for: The effect of helix-inducing constraints and downsizing upon a transcription block survival-derived functional cJun antagonist
Source: Cell Rep Phys Sci. 2022 Oct 19;3(10):101077. doi: 10.1016/j.xcrp.2022.101077 (PMC9582194; doi:10.1016/j.xcrp.2022.101077)
Supplement: Document S2. Article plus supplemental information [file mmc2.pdf]

Cell Reports
Physical Science

CellPress
OPEN ACCESS

Article

The effect of helix-inducing constraints and downsizing upon a transcription block survival-derived functional cJun antagonist

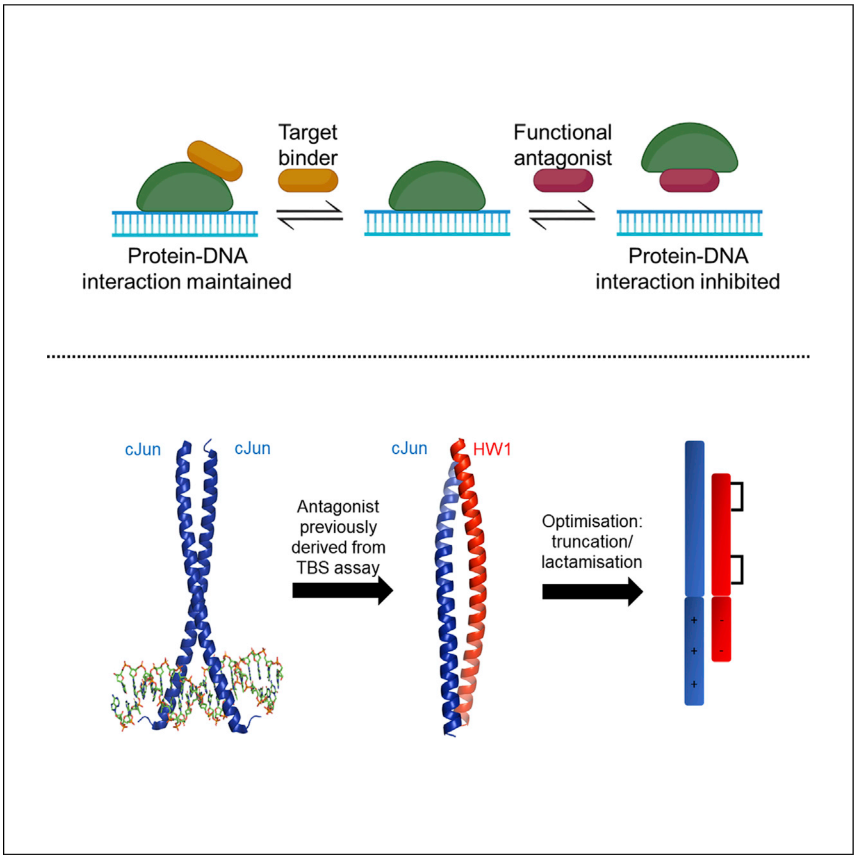

Brennan et al. report that a transcription block survival assay-derived peptide antagonist of validated oncogenic transcriptional regulator cJun is optimized by removing >40% of residues and adding helix-inducing side-chain lactam bridges. The optimized peptide retains function and displays increased serum stability, producing a more drug-like molecule for future applications.

Andrew Brennan, James T. Leech, Neil M. Kad, Jody M. Mason

j.mason@bath.ac.uk

Highlights

A previously derived cJun peptide antagonist has been optimized

Systematic downsizing of HW1 determines the minimal active sequence

Helix-inducing lactam bridges are added to restore efficacy lost from downsizing

HW30 retains activity, is reduced in size, and displays increased serum stability

Brennan et al., Cell Reports Physical Science 3, 101077
October 19, 2022 © 2022 The Author(s).
https://doi.org/10.1016/j.xcrp.2022.101077

Cell Reports
Physical Science

CellPress
OPEN ACCESS

Article

The effect of helix-inducing constraints and downsizing upon a transcription block survival-derived functional cJun antagonist

Andrew Brennan,[1,3] James T. Leech,[1] Neil M. Kad,[2] and Jody M. Mason[1,4,5,*]

SUMMARY

Inhibition of cJun is established as a promising therapeutic approach, particularly in cancer. We recently developed the "transcription block survival" (TBS) screening platform to derive functional peptide antagonists of transcription factor activity by ablating their ability to bind to cognate DNA. Using TBS, we screened a >131,000-member peptide library to select a 63-mer peptide that bound cJun and prevented 12-*O*-tetradecanoylphorbol-13-acetate response element (TRE) DNA binding. Iterative truncation was next combined with a systematic exploration of side-chain cyclization to derive a minimal active sequence. The resulting dual lactamized sequence was >40% smaller and retained low nM target affinity (equilibrium binding constant [K_D] = 0.2 versus 9.7 nM), with 8 residues at the acidic region required for functional antagonism. However, even modest C-terminal truncation resulted in functional loss. The peptide functionally antagonizes cJun (half-maximal inhibitory concentration [IC_{50}] = 13 versus 45 μM) and is considerably more stable in human serum relative to its non-lactamized counterpart and HingeW.

INTRODUCTION

Increasingly, intracellular protein-protein interactions (PPIs) are being targeted by peptide-based modulators for therapeutic effect in a range of diseases.[1–5] In particular, peptides can target broader and flatter PPI surfaces, which were previously disregarded as undruggable because they are typically intractable to smaller molecules. Thus, peptides and their mimetics represent a route to therapy in many diseases, where relevant target proteins are mutated, misfolded, upregulated, or overexpressed and therefore produce detrimental outcomes. To overcome potential shortcomings of peptide therapeutics, a range of strategies have been developed and added to the toolkit to produce more stable and bioavailable molecules.[6–14] Via alteration of the peptide sequence, chemical modification, cyclization, and introduction of non-natural backbone or side-chain components, a wide range of desirable features can be imparted such as improved binding, biostability, and cell penetration. A fundamental step in the development of therapeutic peptides is downsizing toward the smallest functional unit required to retain effective binding to produce molecules that can be efficiently synthesized.[15]

cJun is a member of the activator protein-1 (AP-1) family of dimeric transcription factors that is implicated in a wide range of diseases including cancer, diabetes, and arthritis.[16–19] Constituent AP-1 proteins bind specific DNA recognition sites via their basic-leucine zipper (bZIP) domains. The AP-1 bZIP consists of a leucine zipper (LZ)

[1]Department of Life Sciences, University of Bath, Bath BA2 7AY, UK
[2]School of Biological Sciences, University of Kent, Canterbury, CT2 7NH, UK
[3]Twitter: @drandybrennan
[4]Twitter: @jodymmason
[5]Lead contact
*Correspondence: j.mason@bath.ac.uk
https://doi.org/10.1016/j.xcrp.2022.101077

© 2022 The Author(s).
This is an open access article under the CC BY license (http://creativecommons.org/licenses/by/4.0/).

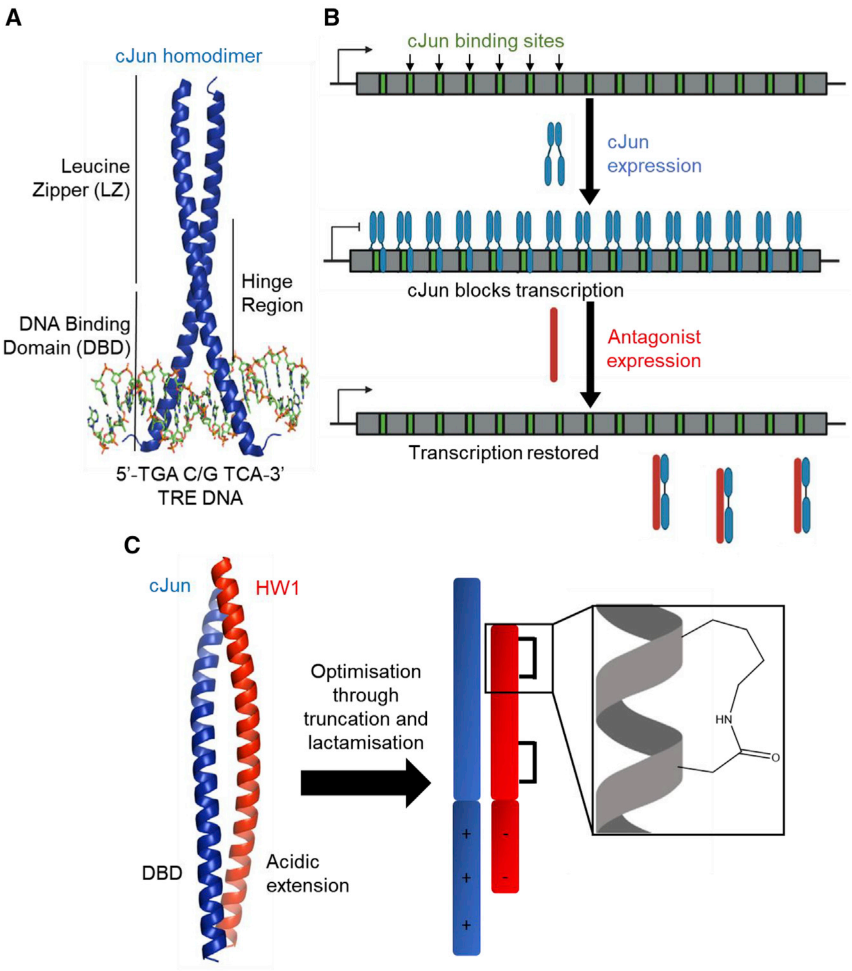

Figure 1. Development of cJun/TRE antagonists via use of acidic extension plus LZ design principles and subsequent optimization via TBS

(A) Crystal structure of a cJun homodimer bound to TRE DNA (PDB: 2H7H).

(B) Cartoon illustrating the TBS assay whereby TRE DNA sites are incorporated into an essential gene to facilitate a cJun-induced transcriptional block, which can only be removed by cJun/TRE DNA interaction antagonists. This produces an assay with cell survival as a readout, which selects for peptide antagonists capable of binding to the target and preventing its interaction with DNA.

(C) AlphaFold-Multimer (run on the Google Colab platform, v.2.1) prediction for the structure of the cJun-HW1 heterodimer, which illustrates the extended coiled-coil interaction between the antagonist with the full length of the bZIP domain, facilitated by the rationally designed N-terminal acidic extension.[20] The TBS winner peptide optimization process then involves truncation and side-chain lactamization.

domain for dimerization and a DNA-binding domain (DBD), which inserts into the major groove, where it recognizes the 12-*O*-tetradecanoylphorbol-13-acetate response element (TRE) via a specific network of electrostatic and hydrogen bond interactions (Figure 1A[20]).[21,22] In this work, we optimize a transcription block survival (TBS) assay-derived hit peptide, HingeW,[23] through sequential truncation combined with systematic screening of side-chain cyclisation.

During TBS, the coding region of the essential gene DHFR is mutated to incorporate TRE sites such that production of cJun inside *E. coli* produces a steric block (Figure 1B). RNA polymerase (RNAP) is then unable to transcribe the DHFR gene,

leading to loss of the ability to reduce dihydrofolate and, ultimately, cell death. Thus, the cJun-induced steric block to RNAP can only be removed upon introduction of a functionally active cJun/TRE antagonist. The growth rate of a particular cell is intrinsically linked to the ability of a peptide library member to remove the transcriptional block and therefore to restore DHFR activity, allowing direct competition between library members until a single TBS winner sequence is selected. The library design template combined a previous library-derived LZ antagonist with a rationally designed N-terminal acidic extension.[24–26] The latter was first proposed by Olive et al. to extend the coiled-coil PPI from the LZ into the DBD, thus preventing DNA binding. The N-terminal design incorporated Leu residues at putative heptad **d** positions and a significant number of negatively charged amino acids via rational replacement of Arg/Lys to form favorable electrostatic interactions with basic residues within the cJun DBD, thus outcompeting the native interaction with TRE DNA. In our study, a central tract of residues across the acidic extension and LZ in this parent peptide sequence were semi-randomized, and the >131,000-member library was screened using the TBS platform, resulting in the selection of HingeW as an optimized cJun antagonist capable of both target binding and, more importantly, ablation of DNA binding.[23]

The binding epitope of HingeW is presented on one side of a single α-helix, and, as such, target binding requires the peptide to adopt this secondary structure. However, as an α-helical peptide is progressively downsized, this tends to reduce the helicity as both the interaction interface and extended internal hydrogen bonding network become reduced and water competes for these interactions, shifting the folding equilibrium toward a random coil. Peptide stapling is a common methodology utilized to increase the α-helicity of peptides by the covalent linking of amino acid sidechains, typically via *i* to *i+4* or *i* to *i+7* linkages.[6–8,11,14,27] For i→i+4 stapling in coiled-coil antagonists, linkages are typically placed on the opposing solvent-exposed face of the helix at **b**-to-**f** (i.e., within one heptad) or **f**-to-**c** positions (i.e., spanning adjacent heptads) to avoid interference with the binding epitope.[28–30] A range of synthetic methodologies have been utilized for this purpose, including lactamization, all hydrocarbon linkers, disulphide bridges, and various thioethers, with one study indicating **b**-to-**f** (K→D) lactam bridges as the most effective at inducing helicity in short alanine pentapeptides.[6–8,14,31] Furthermore, lactamization provides additional benefit in terms of biostability since proteases universally recognize β-strands, with the constraint providing a further steric block, denying access to the backbone.[32] However, sequence-specific effects influence lactamization, and therefore, potential points throughout each peptide must be tested to discover those locations that maximally improve target binding affinity.[30] The process of peptide downsizing combined with the introduction of helix-inducing lactam constraints described here has produced a range of short peptides with a >100-fold range in efficacy and an optimized peptide, which retains nanomolar affinity while removing >40% of the parent sequence (Figure 1C).

RESULTS

Partial truncation of the acidic domain significantly reduces peptide size while retaining activity

The precise nature of the interaction between the cJun DBD and the rationally designed acidic domain of **HW1** is unknown. **HW1** was, therefore, iteratively truncated from the N terminus (**HW2–6**) to investigate the effect on cJun binding and cJun/TRE DNA antagonism (Table 1). Peptide fraction helicity (fH) was determined by quantifying the circular dichroism (CD) signal at 222 nm of peptide-only samples (Figure S1). Thermal denaturation experiments were then used to determine the melting temperature (T_m) of peptide-cJun heterodimers, which serves as an approximate

Table 1. – Antagonist peptide sequences and thermodynamic parameters for their interaction with cJun

	Acidic-ZIP sequence	T_m (°C)	IC_{50} CD (μM)	fH (%)	Truncation
	defgabcdefgabcdefgabcdefgabcdefgabcdefgabcdefgabcdefgabcd				
HW1	MASLEQRAEELARENEELEKEAEELVVEEDVLEEEIEQLEERNYALRKEIEDLQKQLEKLGAPHHHHHH	71.2 ± 1.4	13.4 ± 0.6	27.2	–
HW2	Ac-LARENEELEKEAEELVVEEDVLEEEIEQLEERNYALRKEIEDLQKQLEKL-NH_2	68.5 ± 1.1	16.6 ± 0.5	32.8	NΔ10 CΔ3
HW3	Ac-LEKEAEELVVEEDVLEEEIEQLEERNYALRKEIEDLQKQLEKL-NH_2	67.9 ± 0.9	33.7 ± 2.8	38.3	NΔ17 CΔ3
HW4	Ac-EAEELVVEEDVLEEEIEQLEERNYALRKEIEDLQKQLEKL-NH_2	64.1 ± 1.5	45.4 ± 3.6	45.5	NΔ20 CΔ3
HW5	Ac-LVVEEDVLEEEIEQLEERNYALRKEIEDLQKQLEKL-NH_2	62.8 ± 1.0	78.2 ± 4.1	46.7	NΔ24 CΔ3
HW6	EDVLEEEIEQLEERNYALRKEIEDLQKQLEKLGAP-NH_2	55.1 ± 1.1	129.8 ± 13.0	38.0	NΔ28
HW7	MASLEQRAEELARENEELEKEAEELVVE-NH_2	ND	ND		CΔ35
HW8	Ac-EAEELVVEEDVLEEEIEQLEERNYALRKEIEDLQKQ-NH_2	57.1 ± 0.9	82.1 ± 6.0	37.3	NΔ20 CΔ7
HW9	Ac-EAEELVVEEDVLEEEIEQLEERNYALRKEIEDL-NH_2	44.8 ± 1.2	1,212.9 ± 118.4	32.8	NΔ20 CΔ10
HW10/HW11	Ac-LVVEEDVLEEEIEQLEEKNKALKDEIEDLQKQLEKLY-NH_2	64.3 ± 1.3/67.2 ± 0.8	83.9 ± 6.2/73.5 ± 5.2	45.6/54.2	NΔ24 CΔ3
HW12/HW13	Ac-LVVEEDVLEEEIEQLEERNYALRKEIEDLQKQLEDL-NH_2	62.1 ± 1.4/68.3 ± 0.9	94.6 ± 7.4/69 ± 4.1	43.0/46.8	NΔ24 CΔ3
HW14/HW15	Ac-EAEELVVEEDVLEEEIEQLEEKNKALKDEIEDLY-NH_2	ND/52.5 ± 1.4	1,559.5 ± 143.9/ 118.5 ± 10.9	30.1/35.0	NΔ20 CΔ10
HW16/HW17	Ac-EAEELVVEEDVLEEEIEQLEERNYALRKEIEDLQ-NH_2	53.8 ± 5.3/57.2 ± 0.4	725 ± 62.6/140 ± 14.3	31.2/36.4	NΔ20 CΔ9
HW18/HW19	Ac-KEAEDLVVEEDVLEEEIEQLEERNYALRKEIKDLQDQ-NH_2	56.5 ± 1.6/58.5 ± 1.0	115.0 ± 13.4/92.5 ± 3.4	38.7/40.1	NΔ19 CΔ7
HW20/HW21	Ac-EAKELVDEEDVLEEEIEQLEERNYALRKEIEDLQKQ-NH_2	57.5 ± 1.1/59.4 ± 1.0	100.4 ± 3.9/81.1 ± 5.7	36.9/39.2	NΔ20 CΔ7
HW22/HW23	Ac-EAEELVVEEKVLEDEIEQLEERNYALRKEIEDLQKQ-NH_2	54.9 ± 1.2/64.0 ± 0.8	113.6 ± 10.8/74.9 ± 4.1	37.2/42.6	NΔ20 CΔ7
HW24/HW25	Ac-EAEELVVEEDVLEEEIKQLEDRNYALRKEIEDLQKQ-NH_2	55.5 ± 1.7/60.9 ± 3.6	98.9 ± 5.1/94.4 ± 4.5	37.0/38.7	NΔ20 CΔ7
HW26/HW27	Ac-EAEELVVEEDVLEEEIEQLEEKNKALKDEIEDLQKQY-NH_2	56.2 ± 1.3/58.9 ± 1.0	120.8 ± 17.3/94.1 ± 6.0	36.0/38.5	NΔ20 CΔ7
HW28/HW29	Ac-EAEELVVEEDVLEEEIEQLEERNYALRKEIKDLQDQ-NH_2	55.6 ± 1.2/65.0 ± 1.4	100.8 ± 3.7/52.7 ± 2.0	35.0/43.4	NΔ20 CΔ7
HW30	Ac-EAEELVVEEKVLEDEIEQLEERNYALRKEIKDLQDQ-NH_2	63.2 ± 1.3	44.9 ± 2.1	47.1	NΔ20 CΔ7

Underlining of K,D pairs indicates the lactamization sites with the peptide names and parameters indicated as linear/cyclized. T_m and fH values were determined from duplicate experiments, and IC_{50} values were determined from triplicate experiments. Errors are given as one SD.

CellPress
OPEN ACCESS

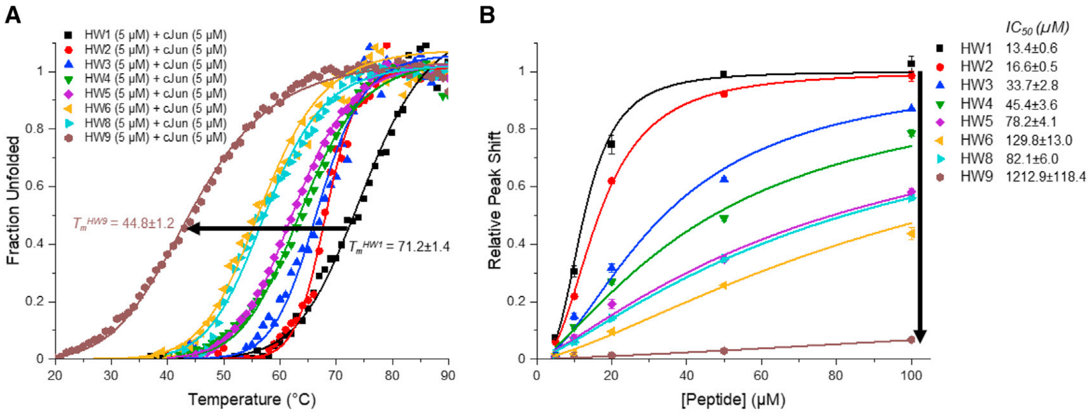


```
cJun(target):     RIKAERKRMRNRIAASKCRKRKLERIARLEEKVKTLKAQNYELASTANMLREQVAQL
A-FosW(parent):   LEQRAEELARENEELEKEAEELEQELDELQAEIEQLEERNYALRKEIEDLQKQLEKL

HW1:              LEQRAEELARENEELEKEAEELVVEEDVLEEEIEQLEERNYALRKEIEDLQKQLEKL
HW2:                     LARENEELEKEAEELVVEEDVLEEEIEQLEERNYALRKEIEDLQKQLEKL
HW3:                            LEKEAEELVVEEDVLEEEIEQLEERNYALRKEIEDLQKQLEKL
HW4:                               EAEELVVEEDVLEEEIEQLEERNYALRKEIEDLQKQLEKL
HW5:                                   LVVEEDVLEEEIEQLEERNYALRKEIEDLQKQLEKL
HW6:                                       EDVLEEEIEQLEERNYALRKEIEDLQKQLEKL
HW8:                               EAEELVVEEKVLEDEIEQLEERNYALRKEIKDLQDQ
HW9:                               EAEELVVEEKVLEDEIEQLEERNYALRKEIKDL
```

Figure 2. Iterative N-terminal truncation of HingeW reduces cJun binding and antagonism

(A) Thermal denaturation profiles for iteratively truncated peptide (5 μM)/cJun (5 μM) heterodimer samples.

(B) CD antagonism data produced by monitoring the shift in a DNA specific peak to provide a direct readout of cJun-DNA binding. Peptides are added at the indicated concentrations to a pre-incubated mixture of cJun (20 μM) and TRE DNA (5 μM). Data points were averaged from triplicate experiments, and error bars indicate one SD. The data were fitted to a Hill equation to determine IC_{50} values. Some higher peptide concentration data points for **HW6** and **HW9** are not shown in this plot for clarity.

measure of target engagement. CD was also used to investigate the functional activity of peptides in antagonizing the cJun/TRE DNA interaction. The TRE-DNA construct used produces a positive CD peak at ~281 nm, which decreases in intensity upon cJun binding in a concentration-dependent manner.[23] cJun does not absorb at this wavelength, which allows a direct measurement of DNA structure, and the shift is only observed for DNA that contains the TRE site (Figure S2). Further, antagonist peptides were shown to have no effect on TRE DNA structure (Figure S3). This provides a clear and direct measurement of the proportion of DNA bound to cJun. As antagonist peptide concentration was increased, the peak shifted to overlay with that of free TRE-DNA (Figure S4). For each peptide, the data were fit to a Hill equation to determine half-maximal inhibitory concentration (IC_{50}) values, specific to these assay conditions, providing a clear comparative measure of antagonism. The same 60 amino acid cJun construct, encompassing the entire bZIP domain, was used for all experiments regardless of antagonist peptide length.

N-terminal truncations in the series (**HW2–6**) sequentially reduced target binding and antagonism, indicating that the full length of the acidic extension contributes to peptide activity (Figure 2; Table 1). However, each truncation in the series resulted in an increasingly large effect on the antagonism per residue removed. Inspection of the iterative truncations from **HW1**→**HW2**, from **HW2**→**HW3**, and from **HW3**→**HW5**, representing full heptad deletions, reveals antagonism decreases of 1-, 2-, and 2-fold, respectively. Further, the final N-terminal truncation investigated (**HW5**→**HW6**), which reduces the peptide down to its LZ only, reduced antagonism 1.7-fold despite only removing four residues. The acidic domain alone (**HW7**) was shown not to bind,

indicating that the LZ domain is an absolute requirement for binding that operates synergistically with the acidic extension of HingeW to bind and antagonize cJun. Removal of the full acidic extension domain (**HW1**→**HW6**) reduced cJun/TRE DNA antagonism 9.7-fold. Previously, we observed a highly significant reduction in peptide activity in the TBS assay from peptides with IC_{50} values similar to that observed for peptide **HW6** (129.8 μM), meaning that **HW6** is unlikely to be unable to fully outcompete cJun/TRE DNA binding. This supports the design rationale of the acidic extension and suggested that **HW5** (IC_{50} = 78.2 μM, 5.8× lower antagonism than **HW1** and 1.7× better than **HW6**) may be considered the maximum viable truncation from the N terminus to be taken forward. Importantly, this extends beyond the LZ toward the DBD and may be important in binding and blocking the target from DNA binding.

N-terminal truncation also produced sequential increases in peptide fH, rising from ~27% for **HW1** to ~47% for **HW5**, indicating that deleted regions have reduced peptide helicity (Table 1). The LZ domain of **HW1** has four point mutations from its parent sequence, FosW, which is known to homodimerize.[24] The negative charge of the acidic extension produces electrostatic repulsion and therefore decreases the propensity to homodimerize as indicated by comparing thermal denaturation curves for the peptides alone where **HW5** displayed a T_m of 41°C, whereas a T_m could not be determined for **HW1** as it was significantly lower, and thus the full sigmoidal transition was undefined. However, further truncation from **HW5**→**HW6** reversed this trend, reducing fH to ~38% while removing one acidic and three hydrophobic residues, leaving the LZ-only domain.

Characterization of HW1

HingeW (**HW1**) was developed to bind across the full cJun bZIP binding surface for more effective functional antagonism of TRE binding, relative to DBD-only or LZ-only cJun inhibitors.[23] The nature of the broad, shallow helical binding surface supports the use of longer peptides such as **HW1**. However, it was unclear whether the full length of the sequence is required to achieve functional antagonism. **HW1** was recombinantly produced and biophysically characterized as an N/C-capped (MAS at the N terminus, GAP at the C terminus) and C-terminally 6xHis-tagged protein construct of 69 amino acids in length, with significant negative charge throughout. These characteristics are particularly unfavorable in a potential therapeutic molecule, even among peptides, and as such, optimization of the sequence has been considered and developed here.

Lactamization of truncated peptides increases helicity and activity

HW5 was optimized by incorporating $i \rightarrow i+4$ (K-to-D) lactam bridges through the use of orthogonal-protecting groups (Lys(Mtt) and Asp(O-2-PhiPr)), which can be selectively deprotected (2% trifluoroacetic acid in DCM) and reacted using typical solid-phase chemistry while the peptide is still attached to the resin. The success of the reaction was confirmed using mass spectrometry (MS) to observe the decreased mass from the loss of a water molecule, compared with the linear unreacted peptide (Figure S5). This intramolecular condensation reaction induces peptide α-helicity by covalently linking residues held in proximity by secondary structure despite their distance in the primary structure. In this work, lactams were only introduced at solvent-exposed **b**-to-**f** or **f**-to-**c** heptad positions to prevent disruption of the binding epitope of the antagonist helix. Point mutations to the sequence were required to incorporate the bridging K and D residues, with both linear (**HW10**, **HW12**) and cyclized (**HW11**, **HW13**) versions of each sequence produced by split batch synthesis.

Cyclized peptides **HW11** and **HW13** increase antagonism relative to the parent linear sequence **HW5** 1.8- and 1.9-fold, respectively, with both increased peptide

CellPress
OPEN ACCESS

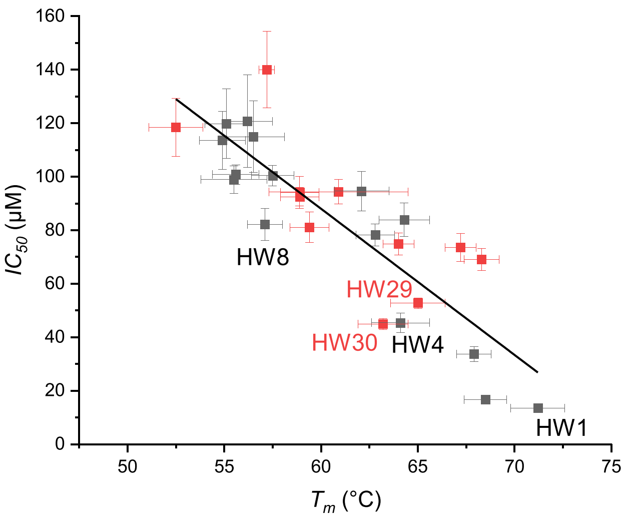

Figure 3. T_m values broadly correlate with IC_{50} values

As the peptide-cJun heterodimer T_m increases, an inverse correlation is observed with IC_{50}, indicating improved cJun/TRE DNA antagonism. Data from **HW7** and **HW14** were not included as values could not be fitted, and **HW9** and **HW16** were not included as their IC_{50} is poorly defined. Data points for linear peptides are shown in gray, and lactamized peptides are shown in red. $r^2 = 0.74$. Error bars indicate one SD.

fH and more favorable target binding (Table 1). It is interesting to note that the binding indicated by T_m values of ~67°C and ~68°C for these peptides do not produce correspondingly low IC_{50} values. By plotting the T_m versus IC_{50} of the peptides in this study, a clear inverse correlation is observed ($r^2 = 0.77$); however, there is variability (Figure 3). **HW2** and **HW13**, for example, have similar T_m values, but **HW2** antagonizes the cJun/TRE interaction 4× more effectively. **HW13** has been truncated by a further 14 N-terminal residues than **HW2**. This supports the importance of the rational design principle used for HingeW whereby inhibition of both domains of the cJun bZIP produces the most effective antagonism, as the majority of the acidic extension has been removed in peptides **HW5** and **HW10–13**, with it all removed in peptide **HW6**. This results in peptides that can be optimized to bind tightly to the cJun LZ but are limited in their ability to functionally antagonize cJun by also blocking the DBD. A crucial point is that any data points below the fitted line have an IC_{50} better than predicted by their T_m and, as such, may be considered the most important in this study, with functional antagonism the goal of this work.

C-terminal truncation has a greater impact on functional peptide activity than does N-terminal

Optimization was therefore best considered by working from **HW4**, which has additional N-terminal negative charge and 1.7× higher antagonism than **HW5**, as the above optimization of antagonism may have been limited by further truncation at the N terminus. This implicates the importance of the region in interacting with the corresponding positively charged cJun DBD surface, as well as inducing helicity and potentially stabilizing the dipole of the molecule with a negatively charged residue at the N terminus.[33–35]

Following this, truncation at the C terminus was considered by the production of **HW8,** which utilized the NΔ20 truncation of **HW4** while also truncating at the C terminus. The removal of four C-terminal residues (10% of the molecule) from **HW 4** to **HW8** reduced antagonism 1.8×, but further truncation at the C terminus to produce

HW9 vastly reduced antagonism 14.8× compared with **HW8** and 26.7× compared with **HW4**. Attempts to optimize **HW9** by lactamization to produce **HW15** and **HW17** were effective in that they significantly improved antagonism; however, they still produce 8.8× and 10.4× reductions in antagonism relative to **HW1** (Table 1). Although these lactamizations produced a much larger impact on the peptides than the other cyclizations in this study, the resulting peptide IC_{50} values remained too high for further consideration.

Systematic lactonization across the peptide sequence identified amenable cyclization sites, leading to the production of the optimized double lactam peptide HW30.

Based on this understanding of the effects of N- and C-terminal truncations, we focused our efforts on **HW8** as a scaffold for further optimization; this peptide is >40% smaller than **HW1** while retaining a high level of functional activity (IC_{50} = 82.1 μM). $i \rightarrow i+4$ (K-to-D) lactam bridges were systematically incorporated at sites throughout the sequence to investigate which regions were most amenable to the helix constraint and which produced improvements in affinity and inhibition. Again, due to point mutations, to introduce the bridging K and D residues, both linear and cyclized peptides were produced. The cJun/antagonist heterodimer change in T_m (ΔT_m) due to lactamization ranged from ~2°C for **HW20→HW21** to ~9°C for **HW28→HW29** (Table 1). It is also useful to compare with the parent sequence, before the substitutions for K and D residues, where the heterodimer ΔT_m ranges from ~1°C for **HW8→HW19** to ~8°C for **HW8→HW29**. cJun/TRE antagonism is the most important measure in this work, however, and only **HW29** produced a significant improvement in IC_{50}, shown to be 1.6× lower than for **HW8** (p = 0.003). Using a lactam constraint, **HW29** was able to restore the reduction in antagonism caused by truncating the four C-terminal residues from **HW4→HW8**. The change in fH of the peptides ranged from ~1% for **HW18→HW19** to ~8% for **HW28→HW29**, and there is a correlation between peptide helicity induced by lactamization at a particular site and the increase in cJun/TRE antagonism observed.

Although the lactamisation within **HW23** did not lead to a significant increase in antagonism (p = 0.24), it did produce the second lowest IC_{50} value of the lactamized peptides in this series, a ~5% increase in peptide helicity, and ~9°C increase in cJun heterodimer T_m relative to its non-lactamized counterpart (**HW22**). Due to the distance between this site and the lactamization site of **HW29**, a double lactamized peptide was produced, **HW30**. The double lactamization produces a peptide that is ~4% more helical, with no significant change in heterodimer T_m but a significant decrease in the value (44.9 ± 2.1 μM, p = 0.02 versus **HW29**), compared with the most effective single lactam peptide **HW29** (Table 1).

Peptide lactamization increases target affinity by increasingly improved enthalpy and an increasingly unfavorable entropic component

A selection of peptides from this work were investigated by isothermal titration calorimetry (ITC) to quantify the thermodynamic parameters of their interaction with target cJun (Figure 4 and S6; Table 2). The determined equilibrium binding constant (K_D) values follow from the CD data above and can be rationalized in terms of the truncation or lactamization they represent. The **HW4**/cJun affinity is 433× lower than **HW1**/cJun due to the removal of 20 residues from the N terminus. Further, removal of four residues from the C terminus to give **HW8** produces a further 12× reduction in affinity. From the maximally truncated **HW8** peptide, we observe 16× and 13× improvements in affinity for **HW23** and **HW29**, respectively, due to the introduction of lactam bridges. Crucially, the double lactamized **HW30** affinity has

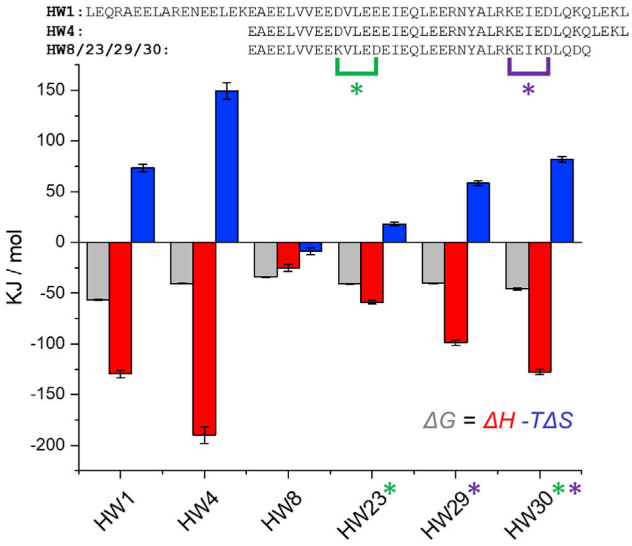

Figure 4. ITC-derived thermodynamic parameters of cJun-peptide interactions, showing a diverse range of binding profiles

ΔG, ΔH, and $-T\Delta S$ are shown in gray, red, and blue, respectively. Experiments were completed at 25°C in duplicate, and error bars show one SD.

increased 113× compared with **HW8** (K_D = 9.69 ± 3.95 nM). All interactions investigated were dominated by the enthalpic component, with a smaller unfavorable entropic contribution, other than for **HW8**/cJun, which is the weakest interaction. The unfavorable entropic component was largest for the **HW30**/cJun interaction and smallest for **HW23**/cJun. This indicates the introduction of lactams produces a more unfavorable entropic component, perhaps a surprising result as helix-inducing lactamization is typically considered to increase affinity through entropic pre-organization. However, the larger increase in the favorable enthalpic component produces an increase in binding affinity due to lactamization. This is likely to be caused by a combination of enhanced intermolecular contacts and ordering that are opposed by unfavorable desolvation effects.

Lactamization increases peptide serum stability

To further explore the effect of truncation and lactamization, **HW1**, **HW8**, **HW29**, and **HW30** were tested for stability in human serum (Figure 5). cFos was included as a linear control peptide, which was the wild-type parental design template on which **HW1** was based prior to optimization.[23–25] The linear peptides (**HW1**, **HW8**, and cFos) were fully degraded over the 3-day experimental time course. In contrast, at this endpoint, the peak intensities of **HW29** and **HW30** were still 31% and 56% of their starting values, respectively. These values demonstrate considerable resistance to protease activity over significant time courses relative to the linear **HW8**

Table 2. ITC-derived thermodynamic parameters cJun-peptide interactions

	Stoichiometry, n	K_D (nM)	ΔH (kJ mol^{-1})	ΔG (kJ mol^{-1})	$-T\Delta S$ (kJ mol^{-1})
HW1	0.93 ± 0.01	0.21 ± 0.03	−129.70 ± 3.65	−56.27 ± 0.75	73.43 ± 3.70
HW4	0.91 ± 0.04	91.1 ± 24.80	−189.95 ± 8.09	−40.50 ± 0.48	149.5 ± 8.10
HW8	0.95 ± 0.09	1,099.50 ± 181.5	−25.06 ± 3.40	−34.10 ± 0.41	−8.95 ± 3.42
HW23	1.20 ± 0.02	69.55 ± 7.83	−59.00 ± 1.68	−40.86 ± 0.28	18.14 ± 1.70
HW29	0.97 ± 0.02	86.55 ± 16.97	−98.74 ± 2.54	−40.42 ± 0.49	58.37 ± 2.59
HW30	1.05 ± 0.01	9.69 ± 3.95	−127.82 ± 2.61	−45.81 ± 1.01	82.01 ± 2.80

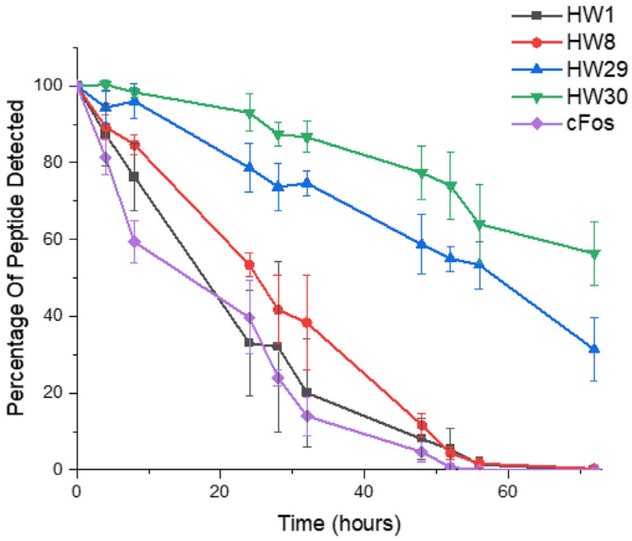

Figure 5. Lactamization results in enhanced serum stability
The quantity of peptide detected by LC-MS is plotted relative to the starting point quantity, demonstrating that the linear peptides (**HW1**/**HW8**/cFos) are degraded more rapidly than when lactamized (**HW29**/**HW30**), with the double lactamized **HW30** demonstrating the highest level of protease resistance. Data points represent averages of three experimental repeats, with the error bars indicating one SD.

counterpart. Moreover, the effect is cumulative, with **HW30** more stable than **HW29** by virtue of the second lactam constraint ($p = 0.039$).

DISCUSSION

We have rationally designed and synthesized a series of peptides based upon the TBS-derived, cJun antagonist HingeW (**HW1**).[23] Our overarching aim was to systematically study the effect of truncation and lactamization on this peptide to enable the production of an optimized antagonist that retains the parent peptides activity while improving its drug-like characteristics. We separated and individually produced the acidic (**HW7**) and LZ (**HW6**) domains of the parent peptide to illustrate their individual contributions to peptide efficacy. **HW6** was shown to antagonize the ability of cJun to bind to TRE DNA ($IC_{50} = 129.8 \pm 13.0$ μM), while the acidic peptide **HW7** did not. However, combining the two regions in **HW1** resulted in enhanced antagonism ($IC_{50} = 13.4 \pm 0.6$ μM). This suggests a mechanism whereby the antagonist LZ binds to the cJun LZ, holding the acidic domain in proximity to the cJun DBD to block the contacts between cJun residues and DNA bases and facilitate higher affinity binding. Further, it is not simply a steric block, since the peptide is highly negatively charged in this region, which will electrostatically repel the like-charged DNA.

The iterative truncation of the acidic domain in peptides **HW1–HW6** showed that the full domain contributed to the peptide's inhibitory effect but that the more N-terminal regions contributed less relative to further truncations in this region. An important consideration in the maximum acceptable truncation is whether a peptide binding to cJun will be able to outcompete TRE DNA. This cJun homodimer/TRE DNA tertiary interaction has been variously established as having a K_D of ~100–200 nM, so maintaining a stronger interaction than this was considered an important benchmark.[22] **HW4** was shown to have a K_D of 91.1 ± 24.8 nM, which places it on the threshold and means that this NΔ20 truncation was considered the maximum acceptable truncation that retained sufficient functional activity. The degree of

CellPress
OPEN ACCESS

truncation at the N terminus that is possible while retaining functional antagonism requires considerations beyond binding affinity, as the acidic domain is crucial for blocking the cJun DBD from binding DNA. Effective antagonism might not require binding to the full length of the cJun DBD; however, peptides **HW5**, **HW6**, and **HW10–13** are no longer able to effectively prevent DNA binding to the cJun DBD, i.e., cJun may still bind to TRE sites in monomeric form, while these antagonist peptides are bound to its LZ domain.[36–38] Specific contacts between particular cJun DBD amino acid side chains and TRE DNA bases are known, so it can be assumed that an antagonist that blocks any of these cJun residues will significantly reduce cJun/TRE binding.[21,22] The four residues added to **HW5** to make **HW4** extend far enough into the cJun DBD to directly block the C-terminal cJun DBD residues known to specifically interact with the TRE site.

Study of C-terminal truncation was less detailed, as the removal of residues here produced larger effects from smaller changes, so fewer stepwise truncations were required. Peptide efficacy was substantially reduced by the CΔ10 truncation, and helix-inducing lactamization was unable to restore the loss to an acceptable degree, as the benefits of truncating fewer residues do not outweigh the activity loss. The CΔ7 truncation, on the other hand, produced losses that could be restored by lactamization and thus were considered a useful optimization, which was further systematically studied.

The thermodynamic parameters of binding observed by ITC show what may be considered an unexpected result in terms of the entropic component. It is usually asserted that introduction of lactam bridges increases binding affinity by pre-organizing the peptide molecule into its helical structure, which can bind to the target with a reduced entropic penalty. The lactamized peptides investigated by ITC have an unfavorable entropic component, with the dual lactam **HW30** having the largest. Further, the length-matched linear peptide **HW8** has a favorable entropic component, so the cyclization-induced shift in entropy is clear. This illustrates that increased target binding affinity from side-chain cyclization can also occur due to improved enthalpic interactions.

Having shown that every truncation of **HW1** produces a peptide with less *in vitro* efficacy, it is important to consider the crucial aspects of a peptide that may make it more suitable for further consideration as a therapeutic. The removal of 27 (in addition to the 6xHis tag) residues to produce **HW30** along with the addition of two lactam bridges produce a peptide that is ~22% more helical and persists in human serum at 56% after 3 days. This is a vast improvement compared with its linear counterparts, which were completely degraded, indicating the potential for favorable pharmacokinetics. Downsizing **HW1** also presented significant practical utility as, due to its length, **HW1** proved to be inaccessible via solid-phase peptide synthesis (SPPS) using standard techniques, with **HW2** and **HW3** producing poor yields. **HW1** was produced as a 6xHis-tagged peptide by recombinant expression and purification from cell lysate, which is a more demanding production process than SPPS. The smaller peptides (**HW4–30**) were all produced in high yield via SPPS, illustrating the time and money that can be saved by downsizing peptides with therapeutic potential.

HW30 is also readily synthesized by SPPS, making it cheaper and quicker to produce. Cyclization of peptides produced recombinantly is either significantly more challenging or impossible, preventing the study of **HW1** lactamization here. Although truncation of **HW1** comes at the cost of a reduction in binding affinity with cJun (0.21 nM), the low nanomolar affinity of **HW30** (9.7 nM versus 1 μM for the linear counterpart) is indicative of an extremely high-affinity interaction, especially when

considered in the context of small-molecule antagonists, which, in inhibiting PPIs of this type, typically display low micromolar affinities at best.[18] HW30 is a highly functionally active inhibitor of cJun/TRE DNA interaction that can be readily produced (facilitating further modifications such as cell penetrance tags) and is highly serum stable. Peptide-based therapeutics are increasingly being developed for the clinic, due to their compelling ability to inhibit PPIs, and as such, downsizing and lactamization are important components in the toolkit to realize these goals.

EXPERIMENTAL PROCEDURES

Resource availability

Lead contact

Further information and requests for resources should be directed to the lead contact, Jody Mason (j.mason@bath.ac.uk).

Materials availability

This study did not generate new unique reagents.

Data and code availability

All data underlying this study are available from the lead contact upon reasonable request.

Peptide synthesis and purification

HW1 was recombinantly expressed and purified as described previously.[23] All other peptides were synthesized using a Liberty Blue microwave peptide synthesizer (CEM) at a 0.1-mmol scale on ChemMatrix Rink amid resin using standard Fmoc solid-phase methodology. Coupling was performed using 5× amino acid, 4.5× PyBOP, and 10× diisopropylethylamine in dimethylformamide (DMF; 5 mL). Deprotection was performed using 20% piperidine in DMF. Peptides were capped at the N terminus by a final reaction with 3× acetic anhydride and 4.5× diisopropylethylamine in DMF for 5 min at 90°C. For lactamized peptides, the relevant K and D positions were orthogonally protected using Lys(Mtt) and Asp(O-2-PhiPr). The side chains of these residues were selectively deprotected by washing the resin with dichloromethane (DCM) ×3, 2% trifluoroacetic acid (TFA) in DCM ×10, DCM 3×, then DMF 3×. The newly deprotected side chains were coupled in PyBOP (1 mL), diisopropylethylamine (1 mL), and DMF (3 mL) for 5 h at 60°C. The resin was dried, and the same reagents added for a second reaction for 16 h at 60°C. Incubation in a cleavage mixture (95% TFA, 2.5% triisopropylsilane, 2.5% H_2O, 10 mL) for 4 h at room temperature cleaved the peptide from the resin and removed side-chain-protecting groups. The resin was removed by filtration, and cleaved peptides were precipitated in diethyl ether at −80°C and centrifuged. This pellet was washed a further four times with diethyl ether before it was dried overnight at room temperature. Peptides were resuspended in 1:1 water:acetonitrile before purification using reversed phase high-performance liquid chromatography (RP-HPLC) with a Jupiter Proteo column (4-μm particle size, 90-Å pore size, 250 × 10 mm; Phenomenex) using a water:acetonitrile gradient (0.1% TFA). Peptide masses and purity (>95%) were verified by electrospray ionization MS.

CD

An Applied Photophysics Chirascan was used for CD measurements, with a 200-μL sample in a 1-mm path length CD cell. Protein/DNA samples were suspended in 20 mM potassium phosphate, 150 mM potassium fluoride, and 2 mM TCEP (pH 7.4). cJun was added to DNA before addition of antagonist peptide, and these samples were equilibrated for 30 min before measurement. For full spectra, three scans between 190 and 260 nm (265–320 nm for DNA-binding experiments) were

collected with a bandwidth of 1 nm and data sampled at a rate of 0.5 s^{-1}. These scans were averaged and converted to molar residue ellipticities (MREs). Thermal denaturation experiments were performed by measuring the ellipticity at 222 nm over a 1°C to 90°C gradient at 1°C increments. Post-melt scans at 20°C confirmed the transitions were reversible, as they overlaid within 10% of the pre-melt scan. The resulting thermal denaturation curves were converted to MREs and fitted to a two-state model, derived via modification of the Gibbs-Helmholtz equation to determine the T_m.[39]

ITC

Peptides were studied by ITC using a Microcal PEAQ-ITC (Malvern Instruments) using an ITC buffer consisting of 20 mM potassium phosphate, 150 mM potassium fluoride, and 2 mM TCEP (pH 7.4). Two-μL injections of antagonist peptide at 25–200 μM were injected into the cell containing cJun at 2.5–20 μM at 25°C. Microcal Control and Analysis software were used to record and analyze the heat change upon addition and fit the data to a one-site binding model to extract the enthalpy change of binding (ΔH) and the K_D, from which the free energy change of binding (ΔG) and the entropy change of binding (ΔS) were calculated.[40] Control experiments involved the injection of the antagonist peptide sample into the cell containing ITC buffer alone to determine the heat of dilution, which was subtracted. Thermodynamic parameters are presented as an average of two independent experiments with errors given as one SD.

Serum stability

Peptide stocks (600 μM) were prepared in water, and 75 μL was added to 1425 μL human serum (Merck) before incubation at 37°C. 100-μL aliquots were removed at designated timepoints and added to 300 μL 3:1 acetonitrile:water and centrifuged (18,000 × *g*, 15 min). The supernatant was analyzed by LC-MS, and the peptide was quantified as the sum of the peaks with the two largest intensities (**HW1**, cFos: 9+, 10+; **HW8**, **HW29**, **HW30**: 3+, 4+).

SUPPLEMENTAL INFORMATION

Supplemental information can be found online at https://doi.org/10.1016/j.xcrp.2022.101077.

ACKNOWLEDGMENTS

J.M.M. is grateful to Cancer Research UK (A26941) and the Medical Research Council (MR/T028254/1). J.M.M. and N.M.K. wish to thank the Biotechnology and Biological Sciences Research Council (BB/R017956/1, BB/R017921/1, and BB/T018275/1).

AUTHOR CONTRIBUTIONS

A.B. conducted the experiments, while all authors contributed to the experimental design. J.M.M. and N.M.K. directed the research. All authors participated in data analysis and writing of the paper.

DECLARATION OF INTERESTS

J.M.M. is an advisor to Sapience Therapeutics. There are no other financial or commercial conflicts to declare.

Received: June 23, 2022
Revised: September 8, 2022
Accepted: September 12, 2022
Published: October 4, 2022

REFERENCES

1. Wang, X., Ni, D., Liu, Y., and Lu, S. (2021). Rational design of peptide-based inhibitors disrupting protein-protein interactions. Front. Chem. *9*, 682675. https://doi.org/10.3389/fchem.2021.682675.
2. Philippe, G.J.B., Craik, D.J., and Henriques, S.T. (2021). Converting peptides into drugs targeting intracellular protein-protein interactions. Drug Discov. Today *26*, 1521–1531. https://doi.org/10.1016/j.drudis.2021.01.022.
3. Buyanova, M., and Pei, D. (2022). Targeting intracellular protein-protein interactions with macrocyclic peptides. Trends Pharmacol. Sci. *43*, 234–248. https://doi.org/10.1016/j.tips.2021.11.008.
4. Lau, J.L., and Dunn, M.K. (2018). Therapeutic peptides: historical perspectives, current development trends, and future directions. Bioorg. Med. Chem. *26*, 2700–2707. https://doi.org/10.1016/j.bmc.2017.06.052.
5. Mason, J.M. (2010). Design and development of peptides and peptide mimetics as antagonists for therapeutic intervention. Future Med. Chem. *2*, 1813–1822. https://doi.org/10.4155/fmc.10.259.
6. Felix, A.M., Heimer, E.P., Wang, C.T., Lambros, T.J., Fournier, A., Mowles, T.F., Maines, S., Campbell, R.M., Wegrzynski, B.B., and Toome, V. (1988). Synthesis, biological activity and conformational analysis of cyclic GRF analogs. Int. J. Pept. Protein Res. *32*, 441–454. https://doi.org/10.1111/j.1399-3011.1988.tb01375.x.
7. Houston, M.E., Gannon, C.L., Kay, C.M., and Hodges, R.S. (1995). Lactam bridge stabilization of alpha-helical peptides: ring size, orientation and positional effects. J. Pept. Sci. *1*, 274–282. https://doi.org/10.1002/psc.310010408.
8. Blackwell, H.E., and Grubbs, R.H. (1998). Highly efficient synthesis of covalently cross-linked peptide helices by ring-closing metathesis. Angew Chem. Int. Ed. Engl. *37*, 3281–3284. https://doi.org/10.1002/(SICI)1521-3773(19981217)37:23<3281::AID-ANIE3281>3.0.CO;2-V.
9. Hamamoto, K., Kida, Y., Zhang, Y., Shimizu, T., and Kuwano, K. (2002). Antimicrobial activity and stability to proteolysis of small linear cationic peptides with D-amino acid substitutions. Microbiol. Immunol. *46*, 741–749. https://doi.org/10.1111/j.1348-0421.2002.tb02759.x.
10. Gentilucci, L., De Marco, R., and Cerisoli, L. (2010). Chemical modifications designed to improve peptide stability: incorporation of non-natural amino acids, pseudo-peptide bonds, and cyclization. Curr. Pharm. Des. *16*, 3185–3203. https://doi.org/10.2174/138161210793292555.
11. Jo, H., Meinhardt, N., Wu, Y., Kulkarni, S., Hu, X., Low, K.E., Davies, P.L., DeGrado, W.F., and Greenbaum, D.C. (2012). Development of α-helical calpain probes by mimicking a natural protein-protein interaction. J. Am. Chem. Soc. *134*, 17704–17713. https://doi.org/10.1021/ja307599z.
12. Agrawal, P., Bhalla, S., Usmani, S.S., Singh, S., Chaudhary, K., Raghava, G.P.S., and Gautam, A. (2016). CPPsite 2.0: a repository of experimentally validated cell-penetrating peptides. Nucleic Acids Res. *44*, D1098–D1103. https://doi.org/10.1093/nar/gkv1266.
13. Moradi, S.V., Hussein, W.M., Varamini, P., Simerska, P., and Toth, I. (2016). Glycosylation, an effective synthetic strategy to improve the bioavailability of therapeutic peptides. Chem. Sci. *7*, 2492–2500. https://doi.org/10.1039/c5sc04392a.
14. Li, X., Chen, S., Zhang, W.D., and Hu, H.G. (2020). Stapled helical peptides bearing different anchoring residues. Chem. Rev. *120*, 10079–10144. https://doi.org/10.1021/acs.chemrev.0c00532.
15. Chen, M., and Li, B. (2012). The effect of molecular weights on the survivability of casein-derived antioxidant peptides after the simulated gastrointestinal digestion. Innov. Food Sci. Emerg. Technol. *16*, 341–348. https://doi.org/10.1016/j.ifset.2012.07.009.
16. Eferl, R., and Wagner, E.F. (2003). AP-1: a double-edged sword in tumorigenesis. Nat. Rev. Cancer *3*, 859–868. https://doi.org/10.1038/nrc1209.
17. Shiozawa, S., and Tsumiyama, K. (2009). Pathogenesis of rheumatoid arthritis and c-Fos/AP-1. Cell Cycle *8*, 1539–1543. https://doi.org/10.4161/cc.8.10.8411.
18. Brennan, A., Leech, J.T., Kad, N.M., and Mason, J.M. (2020). Selective antagonism of cJun for cancer therapy. J. Exp. Clin. Cancer Res. *39*, 184. https://doi.org/10.1186/s13046-020-01686-9.
19. Yung, J.H.M., and Giacca, A. (2020). Role of c-Jun N-terminal kinase (JNK) in obesity and type 2 diabetes. Cells *9*, 706. https://doi.org/10.3390/cells9030706.
20. Evans, R., O'Neill, M., Pritzel, A., Antropova, N., Senior, A., Green, T., Žídek, A., Bates, R., Blackwell, S., Yim, J., et al. (2022). Protein complex prediction with AlphaFold-Multimer. Preprint at bioRxiv. https://doi.org/10.1101/2021.10.04.463034.
21. Glover, J.N., and Harrison, S.C. (1995). Crystal structure of the heterodimeric bZIP transcription factor c-Fos-c-Jun bound to DNA. Nature *373*, 257–261. https://doi.org/10.1038/373257a0.
22. Seldeen, K.L., Deegan, B.J., Bhat, V., Mikles, D.C., McDonald, C.B., and Farooq, A. (2011). Energetic coupling along an allosteric communication channel drives the binding of Jun-Fos heterodimeric transcription factor to DNA. FEBS J. *278*, 2090–2104. https://doi.org/10.1111/j.1742-4658.2011.08124.x.
23. Brennan, A., Leech, J.T., Kad, N.M., and Mason, J.M. (2022). An approach to derive functional peptide inhibitors of transcription factor Activity. JACS Au *2*, 996–1006. https://doi.org/10.1021/jacsau.2c00105.
24. Mason, J.M., Schmitz, M.A., Müller, K.M., and Arndt, K.M. (2006). Semirational design of Jun-Fos coiled coils with increased affinity: universal implications for leucine zipper prediction and design. Proc. Natl. Acad. Sci. USA *103*, 8989–8994. https://doi.org/10.1073/pnas.0509880103.
25. Olive, M., Krylov, D., Echlin, D.R., Gardner, K., Taparowsky, E., and Vinson, C. (1997). A dominant negative to activation protein-1 (AP1) that abolishes DNA binding and inhibits oncogenesis. J. Biol. Chem. *272*, 18586–18594. https://doi.org/10.1074/jbc.272.30.18586.
26. Olive, M., Williams, S.C., Dezan, C., Johnson, P.F., and Vinson, C. (1996). Design of a C/EBP-specific, dominant-negative bZIP protein with both inhibitory and gain-of-function properties. J. Biol. Chem. *271*, 2040–2047. https://doi.org/10.1074/jbc.271.4.2040.
27. Patgiri, A., Jochim, A.L., and Arora, P.S. (2008). A hydrogen bond surrogate approach for stabilization of short peptide sequences in alpha-helical conformation. Acc. Chem. Res. *41*, 1289–1300. https://doi.org/10.1021/ar700264k.
28. Rao, T., Ruiz-Gómez, G., Hill, T.A., Hoang, H.N., Fairlie, D.P., and Mason, J.M. (2013). Truncated and helix-constrained peptides with high affinity and specificity for the cFos coiled-coil of AP-1. PLoS One *8*, e59415. https://doi.org/10.1371/journal.pone.0059415.
29. Baxter, D., Perry, S.R., Hill, T.A., Kok, W.M., Zaccai, N.R., Brady, R.L., Fairlie, D.P., and Mason, J.M. (2017). Downsizing proto-oncogene cFos to short helix-constrained peptides that bind Jun. ACS Chem. Biol. *12*, 2051–2061. https://doi.org/10.1021/acschembio.7b00303.
30. Lathbridge, A., and Mason, J.M. (2019). Combining constrained heptapeptide cassettes with computational design to create coiled-coil targeting helical peptides. ACS Chem. Biol. *14*, 1293–1304. https://doi.org/10.1021/acschembio.9b00265.
31. de Araujo, A.D., Hoang, H.N., Kok, W.M., Diness, F., Gupta, P., Hill, T.A., Driver, R.W., Price, D.A., Liras, S., and Fairlie, D.P. (2014). Comparative α-helicity of cyclic pentapeptides in water. Angew. Chem. Int. Ed. Engl. *53*, 6965–6969. https://doi.org/10.1002/anie.201310245.
32. Tyndall, J.D.A., Nall, T., and Fairlie, D.P. (2005). Proteases universally recognize beta strands in their active sites. Chem. Rev. *105*, 973–999. https://doi.org/10.1021/cr040669e.
33. Pace, C.N., and Scholtz, J.M. (1998). A helix propensity scale based on experimental studies of peptides and proteins. Biophys. J. *75*, 422–427. https://doi.org/10.1016/s0006-3495(98)77529-0.
34. Sali, D., Bycroft, M., and Fersht, A.R. (1988). Stabilization of protein structure by interaction of alpha-helix dipole with a charged side chain. Nature *335*, 740–743. https://doi.org/10.1038/335740a0.
35. Baker, E.G., Bartlett, G.J., Crump, M.P., Sessions, R.B., Linden, N., Faul, C.F.J., and

CellPress
OPEN ACCESS

Woolfson, D.N. (2015). Local and macroscopic electrostatic interactions in single α-helices. Nat. Chem. Biol. *11*, 221–228. https://doi.org/10.1038/nchembio.1739.

36. Kohler, J.J., and Schepartz, A. (2001). Kinetic studies of Fos.Jun.DNA complex formation: DNA binding prior to dimerization. Biochemistry *40*, 130–142. https://doi.org/10.1021/bi001881p.

37. Seldeen, K.L., McDonald, C.B., Deegan, B.J., and Farooq, A. (2008). Evidence that the bZIP domains of the Jun transcription factor bind to DNA as monomers prior to folding and homodimerization. Arch. Biochem. Biophys. *480*, 75–84. https://doi.org/10.1016/j.abb.2008.10.010.

38. Szalóki, N., Krieger, J.W., Komáromi, I., Tóth, K., and Vámosi, G. (2015). Evidence for homodimerization of the c-Fos transcription factor in live cells revealed by fluorescence microscopy and computer modeling. Mol. Cell Biol. *35*, 3785–3798. https://doi.org/10.1128/MCB.00346-15.

39. Mason, J.M., Hagemann, U.B., and Arndt, K.M. (2007). Improved stability of the Jun-Fos Activator Protein-1 coiled coil motif: a stopped-flow circular dichroism kinetic analysis. J. Biol. Chem. *282*, 23015–23024. https://doi.org/10.1074/jbc.M701828200.

40. Wiseman, T., Williston, S., Brandts, J.F., and Lin, L.N. (1989). Rapid measurement of binding constants and heats of binding using a new titration calorimeter. Anal. Biochem. *179*, 131–137. https://doi.org/10.1016/0003-2697(89)90213-3.

Cell Reports Physical Science, Volume *3*

Supplemental information

The effect of helix-inducing constraints and downsizing upon a transcription block survival-derived functional cJun antagonist

Andrew Brennan, James T. Leech, Neil M. Kad, and Jody M. Mason

Supporting Information

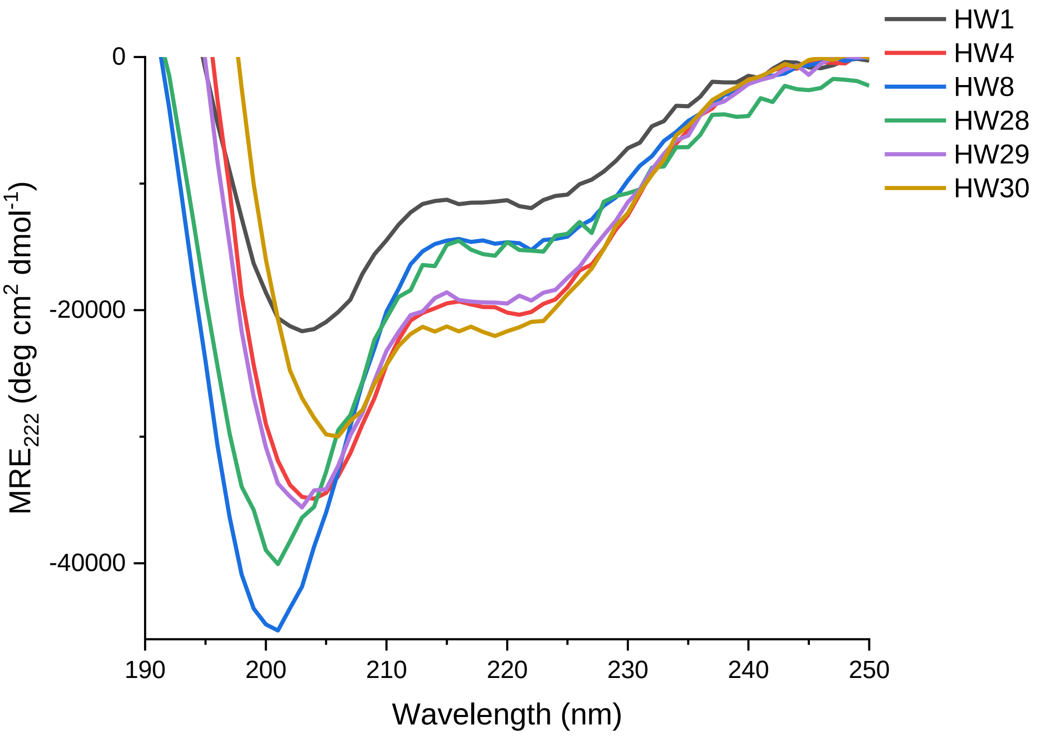

Figure S1 – Circular dichroism spectra showing the changes in peptide structure during the optimisation process. In general, this shows that N-terminal truncation increased helicity, C-terminal truncation decreased helicity and that lactamisation restored lost helicity.

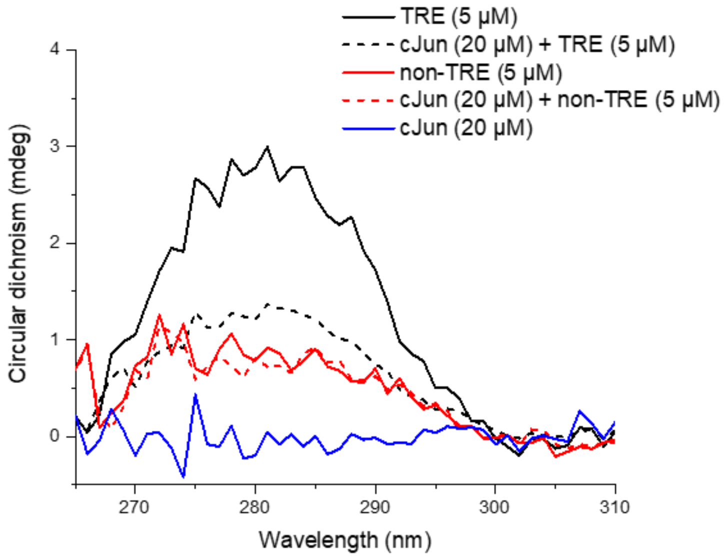

Figure S2 – Circular dichroism spectra showing the TRE site-specific shift in DNA structure caused by cJun binding.

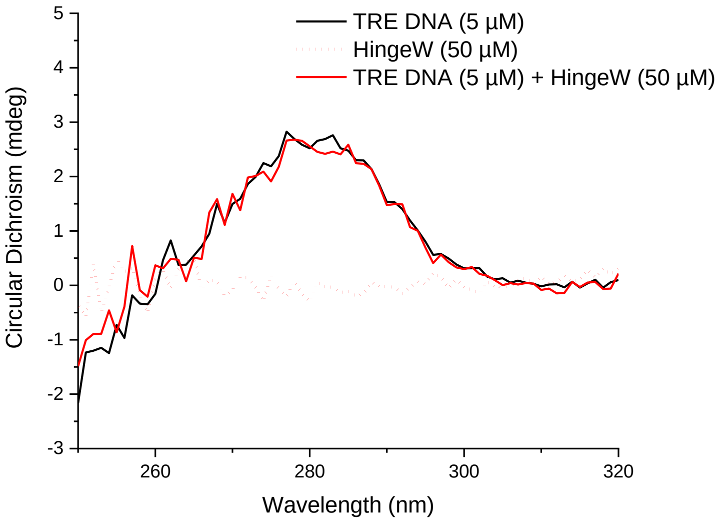

Figure S3 – HingeW does not interact with TRE DNA. Proteins do not absorb in this wavelength range, so the CD signal observed occurs due to the DNA structure, which is not perturbed upon addition of HingeW.

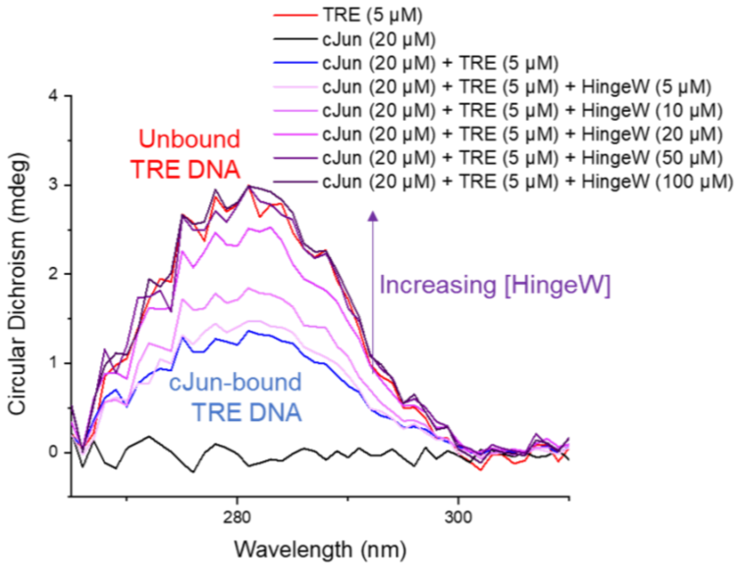

Figure S4 – CD spectra showing the TRE DNA peak shift at 281 nm induced by cJun binding, and its subsequent reversal by HingeW indicative of effective antagonism.

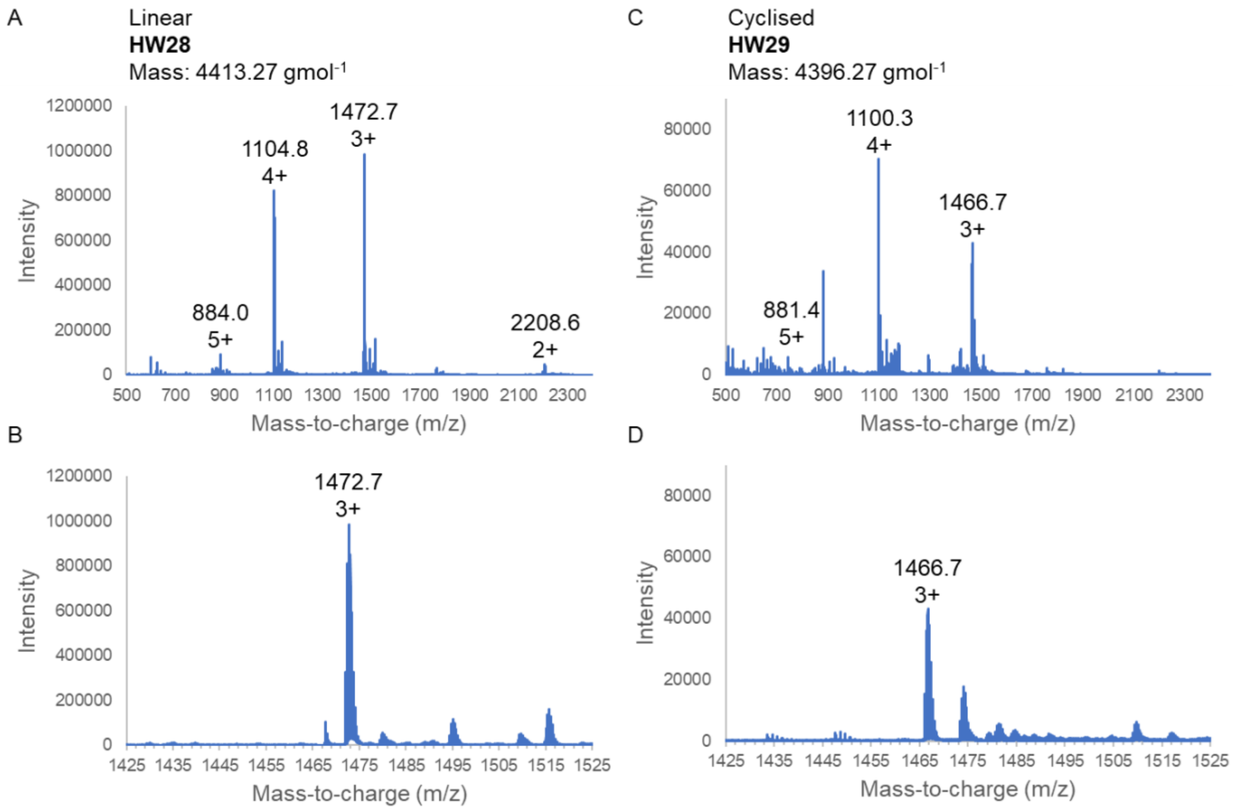

Figure S5 – Mass spectrometry was utilised to confirm peptide identity, to illustrate the shift in mass which occurs due to the loss of water during the lactamisation reaction. (A) MS spectrum of linear peptide HW28 and (B) an expanded region of the same spectrum to highlight the 3+ charge state. (C) MS spectrum of cyclised peptide HW29 and (D) an expanded region of the same spectrum to highlight the 3+ charge state. For this charge state a shift of 6 m/z occurs as predicted, as 6 Da x 3=18 Da (mass of water).

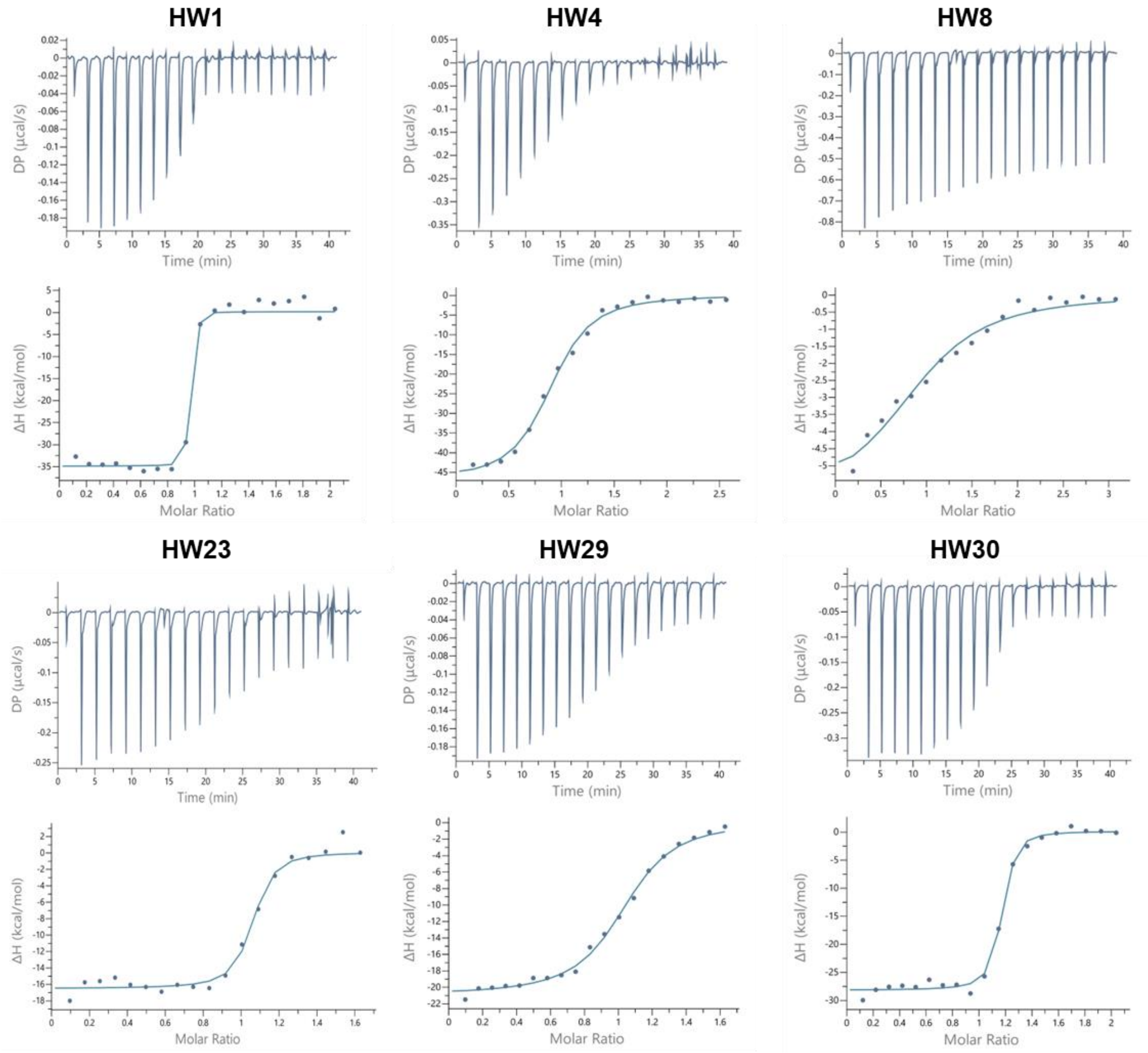

Figure S6 – Isothermal titration calorimetry data for the antagonist peptides binding to cJun. The raw power compensation plot is shown in the upper graph and the integrated data points and single site model fitted line are shown in the lower graph.
